# Multiscale brain development across the human lifespan

**DOI:** 10.1038/s42003-026-10565-6

**Published:** 2026-07-15

**Authors:** Sahar Ahmad, Pew-Thian Yap

**Affiliations:** 1 Department of Radiology, The University of North Carolina at Chapel Hill, Chapel Hill, NC USA; 2 Biomedical Research Imaging Center (BRIC), The University of North Carolina at Chapel Hill, Chapel Hill, NC USA

**Keywords:** Cognitive neuroscience, Neural ageing

## Abstract

Deciphering how the human brain matures and reorganizes across the lifespan remains a central challenge in developmental neuroscience. Understanding the complex developmental processes is essential for elucidating the biological bases of cognition and behavior, as well as the mechanisms underlying aging and neurodegenerative diseases. Neuroimaging has enabled the mapping of nonlinear, age-related changes in brain morphology, microstructure, and connectivity from gestation to senescence. However, we lack a unified understanding of interplay across multimodal neuroimaging measures, structure–function coupling, and the cellular and molecular drivers of network reorganization. In this review, we synthesize current evidence to provide a multiscale MRI-derived account of structural and functional brain development across the human lifespan, highlight key conceptual and methodological gaps, and outline priorities for future research.

## Abstract (web summary)

A comprehensive review on human brain development across multiple scales, from large-scale anatomical structures to microstructural architecture and functional organization across the lifespan.

## Introduction

The human brain undergoes continual development and remodeling throughout the lifespan. This process begins in the third gestational week and continues into adulthood. After reaching maturity, the brain gradually undergoes structural and functional degeneration. A myriad of neurobiological processes orchestrate early brain development, shaping later neurocognitive function and susceptibility to neurodegeneration. Consequently, brain development is a heterogeneous process, characterized by macrostructural and microstructural alterations across the lifespan.

Advances in structural MRI (sMRI), diffusion MRI (dMRI), and functional MRI (fMRI) have enabled increasingly detailed characterization of brain development across the lifespan. However, the field remains challenged by limited longitudinal MRI data, inconsistent computational methodologies, and nonbiological confounds in acquired MRI data. In this review, we highlight current research on brain development across the lifespan, emphasizing how different MRI modalities capture neurodevelopmental processes at multiple spatial and temporal scales. We also discuss recent advances in computational methods that facilitate the processing and analysis of brain MRIs, thereby deepening our understanding of human brain development across the lifespan.

### Brain morphology

Human brain development is characterized by morphological changes throughout the lifespan. Global and regional variations in brain structure contribute to individual differences in cognition and brain function^1^. Charting normative patterns of brain growth across the lifespan is essential for identifying atypical deviations associated with brain disorders. Advances in sMRI, together with the recent availability of large-scale lifespan datasets^2–7^, now enable precise mapping of macroscopic brain development. These efforts have revealed nonlinear, region-specific, and heterochronous patterns of structural maturation and aging, underpinned by cellular and molecular processes such as neurogenesis, synaptogenesis, synaptic pruning, oligodendrogenesis, and myelination^8^.

**Fig. 1:**
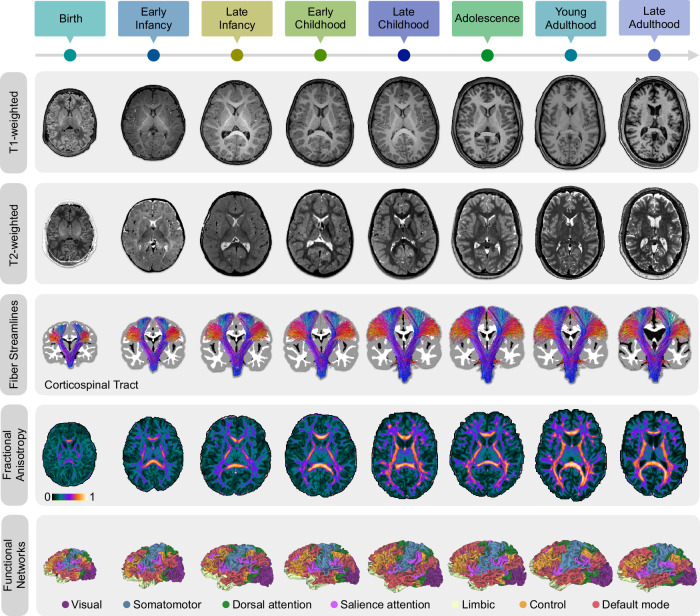
Multiscale brain changes across the human lifespan. T1-weighted and T2-weighted brain MRIs, white matter tracts, fractional anisotropy, and large-scale functional networks of typically-developing individuals between birth and advanced age.

Brain development in early life is marked by rapid increases in brain size, greater cortical folding complexity, and pronounced changes in tissue contrast (Fig. 1). The infant brain reaches  ~ 64% of adult brain volume within the first three months^9^. During the first postnatal year, gray matter volume increases by ~ 97% and white matter volume by  ~ 93% ^10^. Across later development, different brain tissues follow distinct trajectories. Cerebral gray matter volume peaks in middle childhood, whereas subcortical gray matter volume peaks during adolescence^11^. In contrast, white matter volume increases more gradually and continues to rise into young adulthood^11^. Cerebrospinal fluid (CSF) volume increases across the lifespan, with modest expansion in early life followed by accelerated growth in late adulthood. This increase largely reflects brain atrophy with aging, which leads to enlargement of the ventricles and subarachnoid spaces in response to reduction in the overall brain volume^12^. Other brain structures also show characteristic age-related volume changes. Extra-cortical structures, including the subcortex, cerebellum, and brainstem, generally follow inverted U-shaped trajectories, with rapid early life growth followed by gradual decline^13^. The cerebellum grows the fastest, with its volume more than doubling during the first 90 days, whereas the hippocampus grows the slowest, increasing by only 47%^9^. The basal ganglia (caudate nucleus, nucleus accumbens, putamen, and globus pallidus) increase in volume until roughly the first decade of life and then decline linearly with age^13^. In contrast, the thalamus and limbic structures (hippocampus and amygdala) continue to grow into young adulthood, remain relatively stable through midlife, and decline after the sixth decade. One study of individuals aged 5–90 years reported annual gray matter decline rates of 0.51% in the amygdala, 0.314% in the putamen, 0.19% in the caudate nucleus, and 0.155% in the cerebellum^14^. The brainstem reaches its maximal volume at approximately 51 years of age. Analyses of cortical regional volumes using the Desikan-Killiany parcellation further show heterogeneity in developmental timing. For example, the primary sensory regions reach peak volume earliest, during toddlerhood, whereas the association regions peak much later^11^.

Cerebellar lobules also exhibit heterogeneous volumetric changes across the lifespan. Postnatal lobular development is characterized by rapid volume increases during the first year of life, with prolonged growth in higher-order lobules such as Crus I, Crus II, and VIIB, whereas lobule X shows an earlier transition to slowed growth at approximately 200 days after birth^15^. During childhood and adolescence, the greatest volumetric expansion occurs in the corpus medullare^16^. Cerebellar gray matter lobules also display spatially organized developmental patterns along the anterior–posterior axis. The anterior lobe, associated with sensorimotor functions, matures earlier than the posterior lobe, associated with higher-order functions^16,17^. From 20 to 80 years of age, several cerebellar regions exhibit significant age-related volume decline, including parts of the anterior lobe (right I–III and V), posterior lobe (bilateral VI, Crus I, Crus II, VIIB, and IX), the flocculonodular lobe (right X), and vermis X^18^. Another study reported the steepest age-related volumetric decline in the central zone of the cerebellum^19^. Progressive cerebellar volume loss has been linked to functional impairments in both cognitive and motor domains^20–22^.

During early brain development^23^, complex gyrification transforms the initially smooth cerebral surface into a highly convoluted structure. This process occurs across multiple spatial scales: (i) at a global scale, the cerebral surface is divided into two hemispheres; and (ii) at a finer scale, primary, secondary, and tertiary folds progressively emerge. As the brain develops across the lifespan, regions also change in shape and orientation. For example, the narrow sulcal spaces observed in the infant brain widen with aging^24,25^. In addition, cortical gyri and sulci exhibit substantial morphological variability both within and across individuals. Cortical thickness, defined as the vertex-wise distance between the white and pial surfaces, has been extensively studied across the lifespan. The cerebral cortex is relatively thick during infancy, reaching a peak at approximately 1.7 years of age, and then undergoes progressive thinning from childhood through adolescence and adulthood^11,26^. These changes are not spatially uniform but instead follow a gradient along the sensorimotor–association axis^27^. Primary sensory and motor cortices exhibit earlier and more rapid reductions in cortical thickness before childhood, whereas heteromodal and paralimbic cortices mature more gradually and continue to thin into the early 20s^28,29^.

In terms of surface area, the cerebral cortex expands dramatically during the first postnatal year, nearly doubling on average, with particularly pronounced increases in the temporal, parietal, prefrontal, occipital, and sensorimotor cortices^30^. Surface area continues to increase during the second year of life, although at a slower rate, and this expansion persists into early childhood in a region-specific manner. The largest increases in surface area, ranging from 87% to 108%, have been observed in the lingual, fusiform, and rostral anterior cingulate gyri^31^. Overall, cortical surface area shows a protracted developmental trajectory, peaking during late childhood or adolescence and gradually declining throughout adulthood^11,32–34^.

## Structural networks

Structural brain networks provide a systems-level framework for understanding how neural architecture and inter-regional communication in the human brain emerge, reorganize, and decline across the lifespan. These networks are typically constructed by combining dMRI-based tractography, which maps white matter fiber pathways (Fig. 1), with sMRI-based parcellation that delineates distinct brain regions^35^. In this framework, brain regions are represented as nodes and white matter tracts as edges connecting them. The strength of a connection between two regions can be quantified using measures such as streamline count, streamline density, or along-tract microstructural properties^36^. These measures define a weighted adjacency matrix, to which graph-theoretical methods are applied to characterize the organization and properties of structural brain networks^37^.

Leveraging these network construction tools, a growing body of work has characterized nonlinear changes in structural brain networks driven by multiple neurobiological processes, including synaptogenesis, synaptic pruning, axonal growth, myelination, and apoptosis^38,39^. Network strength, local efficiency, and clustering coefficient increase across the lifespan, while global efficiency fluctuates with its peak occurring around 29 years followed by continuous decline through late life^40–42^. Small-worldness demonstrates an inverse pattern, reaching a minimum in young adulthood followed by a nonlinear increase^40^. During adolescence, structural brain network modules become progressively more segregated, driven by strengthening of within-module connections and weakening of between-module connections^43^. These reconfigurations are concentrated at hub edges, enabling networks to achieve greater modular specialization while maintaining efficient global integration^40,44,45^. Wiring distance analyses further highlight the interplay between integration and segregation: although both short- and long-range connections strengthen during development and weaken with aging, the relative contribution of short-range connectivity decreases as the brain matures^46,47^. For further reading on structural networks organization across the lifespan, see Table 1.

**Table 1:** Related papers on structural and functional networks development across the lifespan

Topic	Reference	Key contribution
Structural networks	Huang et al.^165^	Brain networks dynamics from infancy through childhood
	Cao et al.^53^	Review paper on connectome development from infancy through early childhood
	Tymofiyeva et al.^166^	Characterized topological properties of brain networks during early infancy
	Yap et al.^167^	Network topology changes during infancy
	Zhao et al.^168^	Topological organization across the lifespan
	Collin et al.^169^	Review paper on connectome organization across the lifespan
	Puxeddu et al.^170^	Characterized modular organization of brain from childhood to old age
	Riedel et al.^171^	Structural network development from early childhood to old age
	Zalesky et al.^172^	Perspective on connectome mapping in disease
Functional networks	Bero et al.^173^	Age-related changes in spatial and temporal scales of fMRI data
	Wen et al.^174^	Local functional connectivity changes across the adult lifespan
	Luo et al.^175^	Mapped functional connectivity changes along the sensorimotor–association axis from childhood through adolescence
	Sanders et al.^176^	Networks connectivity changes between childhood and adolescence
	Sherman et al.^177^	Development of default mode and executive networks during early adolescence
	Fan et al.^178^	Default mode network development from childhood to adolescence
	Wu et al.^179^	Review on brain connectome changes from gestation through early childhood

## Functional networks

Functional brain networks consist of groups of brain regions that exhibit co-fluctuating activity, typically measured using the blood-oxygen-level-dependent (BOLD) signal in fMRI (Fig. 1)^48^. Regions are considered functionally connected when their BOLD time series exhibit strong statistical dependence, commonly quantified using correlation, coherence, or related measures. Large-scale functional networks are then identified using analytical approaches such as independent component analysis (ICA), clustering or graph-based methods, and seed-based connectivity analyses. Interactions within and between networks are typically examined using functional connectivity (FC) matrices that quantify correlations among regional or network-level time series. These FC measures are often modeled as a function of age to characterize developmental and aging trajectories^49,50^. They are also examined in relation to cognitive and behavioral phenotypes to understand how the progressive reorganization of functional networks supports functional specialization and integration, ultimately shaping behavioral performance across the lifespan^51^.

Functional connectivity within large-scale brain networks undergoes substantial age-dependent changes, beginning in utero and continuing through late adulthood^52^. Functional segregation, the relative dominance of within-network connectivity over between-network connectivity, reflects how distinct and specialized a brain network is based on its connectivity patterns^53^. Previous studies show that functional segregation follows an inverted U-shaped trajectory across the lifespan^49^. It initially increases as large-scale networks rapidly differentiate, and subsequently declines as inter-network coupling strengthens with advancing age, reflecting progressive network dedifferentiation^49,54,55^.

An extensive body of work has examined the emergence, differentiation, and refinement of resting-state functional brain networks across the lifespan^56–58^. These networks include regions supporting primary sensory and motor functions (e.g., visual, auditory, and somatomotor processing), which emerge early in life, as well as higher-order association functions (e.g., cognition, emotion, and memory), which mature more gradually over time^59,60^. Primary networks can be reliably identified in neonates and infants. In particular, somatomotor and auditory networks exhibit relatively stable within-network connectivity during the first postnatal year^61^. In contrast, primary visual networks (V1 and V2) continue to strengthen their within-network connectivity during this period, reflecting ongoing maturation and refinement of visual network organization^61^. Higher-order association networks, including the default mode, frontoparietal, and salience networks, are largely fragmented at birth^61,62^. During the first few years of life, these networks gradually consolidate through the establishment of long-range connections, increased within-network connectivity, and refinement of inter-network boundaries^46,53,63,64^. With advancing age, however, within-network connectivity decreases in several higher-order cognitive networks, including the default mode, cingulo-opercular, and frontoparietal control networks^65^. In contrast, primary sensorimotor and visual networks tend to preserve within-network connectivity but exhibit increased between-network connectivity. In a nutshell, these patterns suggest a shift with aging toward greater functional integration accompanied by reduced functional specialization (i.e., dedifferentiation), which has been linked to age-related cognitive decline^66^. For further reading on functional networks development, see Table 1.

## Tissue microstructure

Macrostructural brain changes are accompanied by continuous microstructural remodeling involving neuronal cell bodies, dendrites, axons, glial cells, and vascular components^67^. These microscopic tissue elements support brain function and can be assessed using dMRI and other quantitative imaging techniques. Metrics derived from diffusion tensor imaging (DTI) and multi-compartment diffusion models, such as neurite orientation dispersion and density imaging (NODDI), enable in vivo characterization of tissue integrity, axonal organization, and microstructural complexity. Using these measures, a growing body of literature has mapped the spatiotemporally heterogeneous maturation and degeneration of brain microstructure across gray and white matter^68–70^.

Fractional anisotropy (FA), which reflects the degree of directional water diffusion, follows a nonlinear lifespan trajectory (Fig. 1). FA increases rapidly during infancy and early childhood^71–73^, continues to rise more gradually through adolescence and early adulthood^74^, and declines with advancing age^75^. These changes are spatially heterogeneous: sensory and motor pathways mature earlier, whereas higher-order frontal–temporal pathways develop later^76^. Increases in FA during development are commonly attributed to myelination^77^, whereas age-related declines likely reflect cumulative myelin loss and glial alterations^78^.

Mean diffusivity (MD), another DTI-derived metric, quantifies the directionally averaged magnitude of water diffusion within tissue and is influenced by factors such as cellular density, membrane integrity, and extracellular space^79^. During early brain development, MD decreases across white matter, reflecting reductions in brain water content and increases in cellular and membrane density that restrict water diffusion^72,80^. As axonal myelination progresses, reduced membrane permeability and extracellular spacing further limit water mobility, leading to continued MD decreases^81^. These reductions persist through adolescence and young adulthood^82,83^. From middle adulthood onward, MD gradually increases, consistent with age-related microstructural degeneration involving myelin degradation and reduced axonal packing^75,84,85^.

Despite its widespread use, DTI provides limited biological specificity because its metrics are influenced by multiple microstructural factors and become unreliable in regions with complex fiber configurations, such as crossing or kissing fibers^86^. To address these limitations, advanced diffusion models adopt biophysically informed frameworks that provide more interpretable tissue properties^87^. One widely used example is NODDI, which decomposes diffusion signals into intracellular, extracellular, and free-water compartments to provide more specific insights into white matter microstructure.

The neurite density index (NDI), derived from NODDI, estimates the intracellular volume fraction and reflects neurite (axons and dendrites) density. Across the lifespan, NDI increases markedly during infancy and childhood, consistent with axonal growth, myelination, and synaptogenesis^87,88^. It continues to increase gradually through adolescence and early adulthood^89,90^, before declining in several white matter pathways after middle adulthood^91,92^. Complementing NDI, the orientation dispersion index (ODI) quantifies the angular variability of neurite orientations within a voxel and reflects the complexity of neurite architecture. Higher ODI values indicate more dispersed or fanned-out structures (e.g., gray matter or the centrum semiovale), whereas lower ODI values reflect highly aligned fiber organization (e.g., corpus callosum)^87^. Studies of ODI report heterogeneous developmental changes during infancy and childhood, with some white matter regions showing increases and others stabilizing early^87–89^. ODI has also been observed to increase in adulthood, although this pattern is thought to reflect age-related myelin remodeling, such as thickening of myelin lamellae and myelin ballooning, rather than ongoing maturation^90^.

Overall, dMRI-derived metrics reveal complex, age-dependent patterns of microstructural remodeling. These changes occur heterogeneously across the brain and follow systematic spatial gradients reflecting the hierarchical organization of white matter development. Such gradients are organized along core-to-periphery, posterior-to-anterior, and inferior-to-superior axes^93–95^. These patterns suggest that primary sensory and motor pathways (e.g., internal capsule, corpus callosum, and occipital regions) mature earlier, whereas higher-order association pathways follow more protracted developmental trajectories consistent with the emergence and refinement of cognitive functions^96^.

## Multimodal data integration

Understanding the developmental trajectories of microstructural and macrostructural properties of white matter tracts is critical for linking morphometry and network organization to the mechanisms supporting efficient communication across the brain. To this end, Schilling et al.^97^ integrated analysis on white matter microstructure, macrostructure, and cortical gray matter features across the lifespan, revealing that pathways develop and age along heterogeneous, spatiotemporally varying trajectories. Volumes of association, commissural, projection, thalamic, and striatal tracts increase during infancy and childhood and begin nonlinear decline in young adulthood. Fiber tracts connecting the frontal or prefrontal cortices are generally thicker than those linking visual or motor cortices. Microstructural indices (FA, radial diffusivity, and intracellular volume fraction) undergo different rates of change across tract types, reflecting tract-specific maturation and relative stability. Quantitative MRI metrics such as R1, R2^*^, magnetization transfer (MT), MT saturation (MTsat), and proton density (PD) provide complementary sensitivity to cortical microstructure, with R1, MT, and MTsat indexing myelin-related macromolecular content, R2^*^ reflecting combined myelin and iron effects, and PD capturing tissue water content^98–100^. Multimodal studies combining these metrics with cortical thickness have revealed multiscale insights into development and aging^101–103^. During childhood, apparent cortical thinning in the visual cortex is largely attributable to increasing intracortical myelination rather than true tissue loss, as reflected by decreases in quantitative T1 and MD in deeper cortical layers^101^. During adolescence and young adulthood, cortical thickness and MT show negative associations^102^. In older adults, R1, R2^*^, and MTsat decline with age in hippocampus, indicating a loss of macromolecular tissue content^103^. These evidence demonstrate the dynamic and regionally heterogeneous interplay between macrostructural morphology and microstructural maturation across the lifespan.

### Morphometric similarity networks

**Fig. 2:**
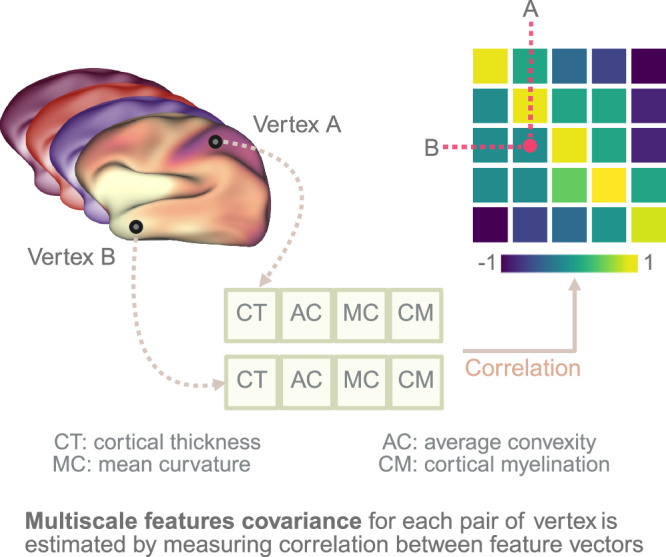
Morphometric similarity networks. Computation of morphometric similarity matrix using cortical features based on geometry and microstructure.

Studying macroscale brain organization in relation to underlying microarchitecture—such as cytoarchitecture, myeloarchitecture, and molecular architecture—requires network models that integrate subject-level multimodal data. Networks derived from tractography and between-subject structural covariance offer complementary views of brain structural organization. However, tractography-based networks are limited by uncertainty in long-range connectivity, while structural covariance networks are constrained by their group-level formulation^104^. To address these limitations, morphometric similarity networks (MSNs) are constructed using subject-level, multiscale cortical features, including cortical thickness, surface area, DTI metrics, and the T1w/T2w ratio (Fig. 2)^104,105^. This framework enables the integration of macrostructural organization with microstructural, cognitive, and transcriptomic domains across the lifespan^106,107^. MSNs have also been used to investigate deviations in cortical connectivity associated with neurological conditions^108^.

The multimodal MSNs examined using combined microstructural and morphological brain features of neonates revealed that MSNs exhibit coherent, symmetric modular architecture aligned with known cytoarchitectonic and functional systems, while undergoing rapid age-related refinement in inter-regional similarity over the perinatal weeks^109^. During adolescence, MSN reorganization is found to be regionally heterogeneous: paralimbic regions, including the insula and cingulate, show increasing morphometric similarity with age, whereas much of the neocortex exhibits declining similarity^110^. These divergent trajectories underscore adolescence as a critical window during which cytoarchitectonically distinct cortical zones undergo unique patterns of structural differentiation and integration.

Further investigations of MSNs in adolescents and young adults reveal a modular organization that closely aligns with known cortical cytoarchitectonic maps, validating biological underpinning^105^. Regional MSN property (edge weights) is associated with cortical gene expression patterns enriched for neuronal functions, while MSN nodal degree is linked to inter-individual differences in cognitive performance. Subsequent work extended this approach across the lifespan to investigate how morphometric similarity explains age-related reorganization of cortical architecture^104^. Using multimodal data from individuals aged 8 years and above, it has been demonstrated that global morphometric dispersion increases with age, where substantial changes occur from childhood to adolescence and renewed increase in late adulthood. At the region level, secondary sensory cortices exhibited age-related decreasing dispersion, consistent with progressive morphometric integration, whereas dorsal prefrontal and insular regions showed increasing dispersion with age, indicative of increasing morphometric segregation from the rest of the brain. The within-network dispersion increases with age in all the networks with differential developmental timing and the between-network dispersion also undergoes nonlinear changes across the lifespan. The developmental and aging patterns of global dispersion were linked to cortical gene expression profiles and to neurotransmitter systems, including acetylcholine, dopamine, and glutamate, highlighting molecular substrates of large-scale structural reorganization. A recent large-scale lifespan study characterized MSNs derived from cortical thickness, surface area, gray matter volume, mean curvature, and sulcal depth, revealing systematic reorganization of network architecture from birth through advanced aging^106^. The global mean network strength decreased through adolescence, while global variance increased. The network strength of the long-range and short-range connections increased, while mid-range connection remained stable. With increasing modularity and small-world organization from birth to early adulthood, these findings demonstrate continuous optimization of integration and segregation of morphometric connections across the lifespan.

### Structure–function coupling

By integrating multiple morphometric features into a single, individual-level representation, MSNs offer a low-dimensional summary of cortical architectural organization that is appropriate for linking structure to function. Another way to understand this mapping is through structure–function coupling (SFC) that measures the correspondence between regional structural similarity and functional connectivity or activation patterns^111^. Lifespan analyses reveal that local coupling is strongest in unimodal sensory and motor networks and weaker in higher-order association networks, following age-related changes: coupling in sensory networks declines across development and aging, whereas higher-order networks largely maintain their structural-functional alignment^112^. These findings highlight the spatial and temporal heterogeneity of structure–function relationships, emphasizing that different cortical systems follow distinct developmental and aging trajectories. Study spanning adolescence (8–23 years) has demonstrated that SFC supports functional specialization and cognitive development^113^. Individual differences in SFC are linked to executive performance, where higher coupling in the rostrolateral prefrontal cortex is associated with better executive function and accounted for age-related improvements in executive ability. Another study examined SFC in children and young adults across default mode, salience, and central executive networks and found that structural and functional connectivity in regions involving the right fronto-insular cortex and dorsolateral prefrontal cortex are stronger in adults and less developed in children^114^. This suggests that SFC shows increasing prominence over the course of development, as white matter pathways undergo structural maturation to support robust functional connectivity in the adult brains. A recent study investigated SFC during infancy and early childhood and found that regional patterns of SFC are similar to those observed during adulthood^115^. From year 1–6, most cortical regions experienced a decrease or no change in SFC, while some regions in the frontoparietal executive and visual networks showed significant increase in SFC. Some studies have also analyzed SFC in typically and atypically developing individuals^116,117^. For instance, SFC development in children with ADHD exhibited weaker coupling in the superior temporal gyrus, prefrontal cortex, and inferior parietal cortex^117^. The rate of coupling strength increased in ADHD, while no change was observed for the healthy cohort.

## Methodological advances

**Fig. 3:**
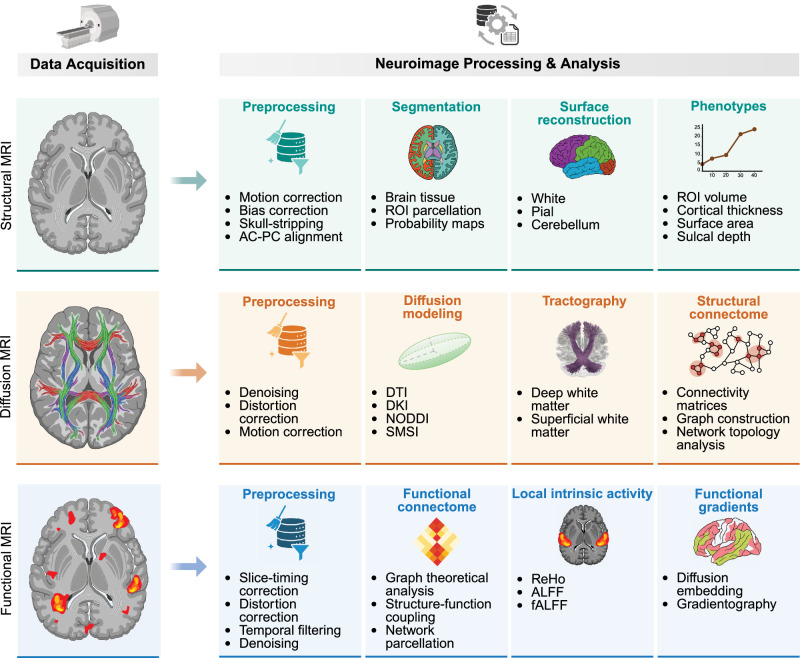
Neuroimage processing and analysis. Overview of computational methods used for processing and analyzing multimodal brain MRIs. DKI diffusion kurtosis imaging, SMSI spherical mean spectrum imaging, ReHo regional homogeneity, ALFF amplitude of low-frequency fluctuations, fALFF fractional ALFF. Created in BioRender. Ahmad, S. (2026) https://BioRender.com/6i4ojfo.

Accurate characterization of brain development across the lifespan depends on computational tools (Fig. 3) that are robust to age-related dynamic brain changes in tissues contrast, brain morphology, and underlying tissue microarchitecture, as well as to MRI noise and bias introduced by motion, acquisition protocols, scanner differences, and field inhomogeneities. Advanced neuroimage processing techniques should be developed to overcome these challenges arising from both biological and technical sources of variability, such that reliable and consistent neuroimage analyses can be conducted across different life stages. Many lifespan neurodevelopmental studies use a heterogeneous set of age-specific computational pipelines (Table 2) to process MRIs across different developmental stages. Although this strategy enables large-scale analyses^11,49,106^, it introduces methodological inconsistencies because each tool is optimized for a narrow age range and incorporates age-specific anatomical priors, algorithmic assumptions, and parameterizations that are not generalizable across different life stages. These inconsistencies can introduce artificial discontinuities in imaging derived phenotypes at transitions between life stages, potentially distorting true growth trajectories and precluding the detection of developmentally critical milestones and inflection points across the lifespan.

**Table 2:** Computational methods for structural MRI (sMRI), diffusion MRI (dMRI), and functional MRI (fMRI) across different life periods

Modality	Tool	Life Period	Functionality
sMRI	MANTiS^180^	Neonatal	Tissue segmentation
	dHCP Pipeline^4^	Neonatal	Anatomical parcellation; cortical surface reconstruction
	BIBSNet^181^	Early infancy	Anatomical parcellation
	Infant FreeSurfer^182^	Infancy	Anatomical parcellation; cortical surface reconstruction
	iBEAT V2.0^183^	Neonatal–early childhood	Tissue segmentation; cortical surface reconstruction
	FreeSurfer^184^	Childhood–adulthood	Anatomical parcellation; cortical surface reconstruction
	BrainSuite^185^	Childhood–adulthood	Anatomical parcellation; cortical surface reconstruction
	CAT12^186^	Childhood–adulthood	Voxel-based morphometry; surface-based morphometry
	FSL (BET/FAST/FLIRT)^187^	Childhood–adulthood	Skull-stripping; tissue segmentation; linear image registration
	NeuroVerse^118^	Birth–adulthood	Anatomical parcellation; cortical surface reconstruction
	uBrainSurf^119^	Birth–adulthood	Cortical surface reconstruction
	BrainParc^120^	Birth–adulthood	Anatomical parcellation
dMRI	TRACULInA^188^	Infancy	Tractography
	TractSeg^189^	Adulthood	White matter tract segmentation
	RecoBundles^190^	Adulthood	White matter tract segmentation
	TRACULA^191^	Adulthood	Tractography
	MRtrix3^192^	Infancy–adulthood	Tractography; structural connectome construction; dMRI registration
	DIPY^193^	Infancy–adulthood	Diffusion modeling; tractography; denoising
	QSIPrep^194^	Childhood–adulthood	dMRI preprocessing
	DSI Studio^195^	Infancy–adulthood	Tractography; connectivity analysis
fMRI	fMRIPrep^196^	Childhood–adulthood	fMRI preprocessing
	XCP-D^197^	Childhood–adulthood	fMRI postprocessing; functional connectivity; denoising
	CONN Toolbox^198^	Adulthood	Functional connectivity
	FSL (MELODIC/FSLNets)^187^	Infancy–adulthood	Voxel- and node-based functional connectivity
	AFNI^199^	Infancy–adulthood	fMRI data analysis
	BrainSpace^200^	Infancy–adulthood	Cortical gradients

To resolve these issues, recent efforts have focused on developing lifespan computational tools^118–120^ that adapt to age-related anatomical and contrast changes within a unified framework, providing consistent regional definitions across development and aging, and reducing nonbiological artifacts for faithful characterization of brain growth across the lifespan. A critical first step in neurodevelopmental studies is skull-stripping or brain extraction, which underpins almost all subsequent neuroimage processing and analyses. The latest work has introduced a deep learning skull-stripping framework that incorporates age-specific atlases as anatomical priors to separate brain from nonbrain tissues in MRIs across the lifespan^121^. Validated across large-scale multi-site datasets, this approach improved robustness to age-dependent anatomical variation and scanner heterogeneity, yielding biologically plausible estimates of brain volume from development through aging. Subsequent to accurate brain extraction, segmentation is performed to delineate cortical, subcortical, and infratentorial structures (brainstem and cerebellum)^122,123^ that are further used for the quantification of region-specific morphology and microstructure, construction of brain networks, and investigation of functional connectivity. One such method, called BrainSMM^124^, introduced a metadata-driven segmentation framework that uses text-based prompts to guide representation learning based on participant’s attributes, such as age and sex. This approach enables consistent delineation of brain regions across different life periods. To mitigate variability in clinical MRIs, domain-randomized deep learning networks trained on synthetic data have facilitated robust anatomical segmentation across heterogeneous imaging protocols, artifacts, and populations^125^. While segmentation traces anatomical regions, surface-based analysis requires 3D representation of the cortical ribbon. The reconstructed cortical surfaces allow studying multidimensional geometric features that are more sensitive to subtle brain changes than volumes^126,127^. In contrast to conventional cortical surface reconstruction pipelines optimized for specific age ranges^128^, recent lifespan reconstruction frameworks adapt to age-related changes in cortical geometry, folding complexity, and gray–white matter contrast^119,129^.

The processing of dMRIs is essential for mapping white matter pathways supporting inter-regional communication^130^. By modeling anisotropic water diffusion, dMRI enables reconstruction of white matter tracts, extending neuroimage analyses from regional morphometry to network connectivity^131^. Several tractography approaches tailored to specific age groups have been developed so far^132–136^, with infant-specific methods designed to accommodate low anisotropy, ongoing myelination, small brain size, and partial volume effects^137,138^. The white matter tracts are typically quantified using microstructural and morphometric measures to characterize developmental and aging-related changes along and across tracts^139,140^. The tractography-based measures are also critical for examining how white matter maturation supports the emergence and reorganization of functional brain networks^112,141^.

The lack of unified lifespan processing approaches results in neuroimaging data being confounded by nonbiological variability introduced by scanners’ hardware and software, imaging protocols, and study designs. This challenge is further exacerbated in mega-analyses of neuroimaging derived phenotypes, where large-scale multi-site datasets are aggregated to increase statistical power and lifespan coverage. Harmonization methods have therefore become essential for disentangling nonbiological artifacts from true brain measures^142–145^. These statistically-grounded techniques remove site- and scanner-related confounds and enable accurate growth modeling across populations.

## Critical gaps

Despite substantial advances in mapping the multifaceted trajectories of brain development and aging, our knowledge remains limited with respect to the emergence and remodeling of brain structure and function and their co-development across the lifespan, and regarding the underlying cellular and molecular processes that drive neural reorganization. Although large-scale, multi-site studies have characterized age-related variations in brain morphology, microstructure, and network connectivity, much of the findings stemmed from cross-sectional data, hence, limiting insights into true longitudinal changes. Adding to these constraints is the use of non-standardized methodological approaches, optimized for specific life stages, that introduce inconsistencies across phenotypes and hinder precise identification of key developmental milestones. A notable gap in lifespan brain development research is the limited integration of structural and functional brain metrics with behavioral and cognitive outcomes. Most existing studies focus on restricted age ranges, often infancy, childhood, or older adulthood, which constrains our understanding of how dynamically evolving brain networks scaffold cognitive abilities, emotions, and behavior across the lifespan. Additionally, disproportionate attention is directed toward understanding cortical development, often overlooking the growth characteristics and functional roles of extra-cortical structures such as the subcortex, brainstem, and cerebellum. These structures undergo remarkable multiscale changes across the lifespan and are integral to cognition, emotion, and motor control. Their relative underrepresentation in brain–behavior studies further limits a systems-level understanding of how distributed neural circuits support behavioral changes across the lifespan.

## Forward-looking perspectives

Future progress in understanding brain development and aging across the lifespan will require sustained efforts in longitudinal neuroimaging studies spanning extended life periods. Recent large-scale initiatives, such as the HEALthy Brain and Child Development (HBCD) study^3^, the Adolescent Brain Cognitive Development (ABCD) study^2^, the NKI Rockland Sample^146^, the Mapping Interindividual Variation in the Aging Connectome (MIVAC) study^147^, and the Chinese Color Nest Project^148^, provide unprecedented opportunities for characterizing within-individual changes in brain structure and function, extending the knowledge gained from cross-sectional data. Realizing the full potential of these datasets will also require the development of computational frameworks capable of processing and modeling neuroimaging data consistently across the entire lifespan. Such tools should be openly shared to promote transparency, reproducibility, and cumulative scientific progress.

**Fig. 4:**
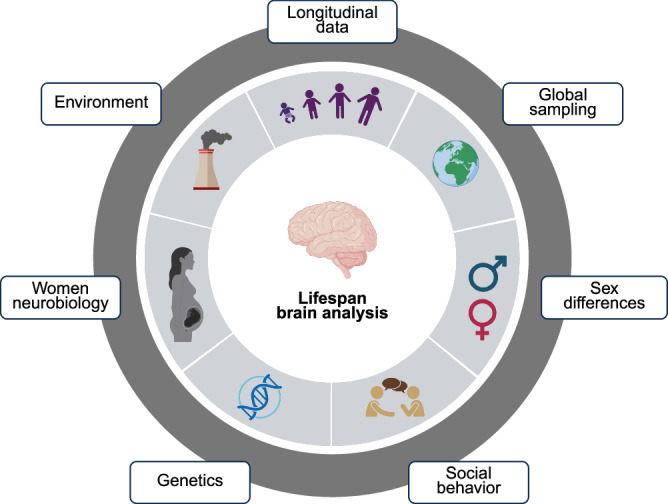
A roadmap for future lifespan brain research. Conceptual overview of research priorities and opportunities for advancing the field. Created in BioRender. Ahmad, S. (2026) https://BioRender.com/6i4ojfo.

It is also important to ensure diversity and inclusivity in study populations. Collecting data with adequate representation of women, individuals from socioeconomically disadvantaged backgrounds and from low- and middle-income countries is critical for understanding how social, environmental, and cultural factors shape brain development and aging (Fig. 4). Broadening the demographic scope of lifespan neuroimaging research will help develop the models of brain organization that are biologically plausible and globally generalizable. Future research in understanding brain plasticity will require large-scale epidemiologically informed studies that integrate MRI data with genetic, environmental, lifestyle, behavioral, and social measures across the lifespan^149,150^. Multivariate analyses of these combined data will clarify how biological and social determinants interact to shape brain development and long-term health outcomes^151–153^. Such integrative approaches will help identify risk factors for cognitive decline and neurological diseases, and delineate age-specific windows of heightened vulnerability to these conditions over the lifetime.

Building upon previous works demonstrating that white matter microstructure provides a structural scaffold for the functional organization of large-scale brain networks^154–157^, future research should expand these findings across the lifespan to establish mechanistic links between tissue microstructure and the emergence and development of functional networks. Integrating diffusion MRI or quantitative MRI measures to functional connectomics may elucidate how micro-level tissue properties, such as neurite density, myelin, tissue integrity, conduction velocity, etc., underlie network segregation and integration, and may also provide a basis to understand functional dysconnectivity in neurological conditions.

A key future direction is the adoption of a lifespan perspective to better understand the links between early brain development and late-life neurodegeneration. Recent findings suggest that certain neurodevelopmental and neurodegenerative disorders share overlapping biological mechanisms, indicating that early developmental disruptions may predispose the brain to later neuropathology^158^. For instance, individuals with Down syndrome exhibit increased risk for autism spectrum disorder early in life^159^ and are also highly susceptible to Alzheimer’s disease and other dementias later in adulthood^160^. These observations suggest that early-life biological vulnerabilities may shape trajectories of brain aging and cognitive decline. Consequently, longitudinal studies spanning from birth through late adulthood are critical for identifying developmental deviations that may predict later neurodegenerative risk. In this context, deep learning models trained to capture lifelong SFC or dynamics of MSNs offer a predictive framework for the identification of brain disorders^161–163^. To this end, graph convolutional networks can integrate structural and functional connectivity, network topology, and nodal features to learn normative patterns of brain organization and the trained models can be used for disease classification^164^. Such lifespan approaches will enable researchers to uncover shared mechanisms underlying co-occurring neurological conditions, to identify earlier diagnostic markers, and to develop preventive interventions that target disease processes decades before clinical symptoms emerge.

## Conclusion

In this review, we examined human brain development across the lifespan, highlighting how different neuroimaging modalities capture complementary aspects of brain changes. By integrating multimodal neuroimaging information, it becomes feasible to map brain growth and organization across multiple spatial and temporal scales. We also discussed recent methodological advances that facilitate the computational processing and analysis of neuroimaging data, facilitating mapping brain development from both cross-sectional and longitudinal datasets across diverse age groups. Continued advances in multimodal neuroimaging and computational and analytical approaches will further enhance our ability to map how the human brain develops and changes across the lifespan, ultimately providing a more comprehensive understanding of the mechanisms that shape brain structure and function.

### Reporting summary

Further information on research design is available in the Nature Portfolio Reporting Summary linked to this article.

## Supplementary information

Reporting Summary

Transparent Peer Review file
