# Supplementary material for: Multiscale brain development across the human lifespan
Source: Commun Biol. 2026 Jul 15;9:965. doi: 10.1038/s42003-026-10565-6 (PMC13373200; doi:10.1038/s42003-026-10565-6)
Supplement: Supplementary file 2 — Transparent Peer Review file [file 42003_2026_10565_MOESM2_ESM.pdf]

Peer Review File

Multiscale Brain Development Across the Human Lifespan

Corresponding Author: Dr Sahar Ahmad

This file contains all reviewer reports in order by version, followed by all author rebuttals in order by version.

Version 0:

Reviewer comments:

Reviewer #1

(Remarks to the Author)
This is a timely and comprehensive review of lifespan brain development. The authors have done a commendable job synthesizing a vast literature across macrostructural, microstructural, and network scales, with particularly strong sections on morphometric similarity networks (MSNs) and structure-function coupling. The manuscript is logically structured, and the figures provide excellent orientation to the primary literature. However, before it is fully suitable for publication, there are a few conceptual and scoping issues that need to be addressed to enhance the manuscript's precision and theoretical depth.

1. The title implies a modality-agnostic review of brain development, yet the content is strictly focused on magnetic resonance imaging (sMRI, dMRI, fMRI), which the authors appropriately note in their Keywords. However, the broader developmental connectomics field relies heavily on electrophysiological and optical imaging (MEG, EEG, fNIRS). This is especially critical at the early temporal extremes of the lifespan (neonates and infants), where MRI faces significant logistical and ethical constraints.
The authors should either explicitly narrow the review's scope in the title (e.g., adding a subtitle like "An MRI Perspective") and introduction, or add a brief discussion acknowledging the complementary roles of these other modalities in filling the gaps left by MRI.
2. While the perspectives section correctly identifies key future directions, it currently reads somewhat like a high-level checklist. Weaving in the following cutting-edge areas would significantly strengthen its theoretical and clinical impact:

Microstructure-to-Function Maturation: The manuscript thoroughly reviews microstructural changes (e.g., DTI/NODDI) and macro-level structure-function coupling, but the transition between these scales feels slightly disjointed. In the perspectives, it would be highly beneficial to discuss how specific micro-level alterations (e.g., neurite density, myelination trajectories) directly constrain or support the maturation of functional networks (e.g., DMN). This would better unify the multi-scale integration theme.

Mechanisms of Individual Differences & Plasticity: Figure 4 excellently highlights "Genetics," "Environment," and "Social behavior." However, the accompanying text primarily frames these within the context of "global sampling" and "demographic inclusivity." The authors should expand on this to explicitly discuss how we can use multimodal lifespan data to understand the sources of individual variance and brain plasticity, moving beyond just having diverse samples to actually modeling the mechanistic impacts of genetics and early experiences.

Bridging Methodological Advances (AI) with Clinical Translation: The authors highlight deep learning in the "Methodological Advances" section for image processing (e.g., skull-stripping, segmentation). They also end the review discussing the prediction of late-life neurodegenerative risk from early developmental deviations. These two themes should be connected. It would be worth noting how AI/deep learning could leverage these multi-scale normative atlases (like MSNs or SFC) to establish precise, personalized biological markers for the early identification of neurodevelopmental and neurodegenerative risks.

Reviewer #2

(Remarks to the Author)
This is an ambitious narrative review of brain development across the lifespan. The manuscript is well written and

remarkably comprehensive. The encyclopedic review of the literature will serve as a resource for the developmental imaging community as it extends from the first decade of this century to recent milestones and fundamental methodological papers. The synthesis is brief but authoritative.

A few minor points:
In line 31, review does not need to be capitalized.
In line 172, “In nutshell” should be “In a nutshell…”
In line 278, “map” should be “maps”
In line 349, “Latest work has introduced …” is incomplete. It could be “The latest work…”
In 359, the authors refer to gender. Gender, the psychological construct, is rarely the focus of neuroimaging studies, which instead dichotomize on biological sex.
In 371, in “extending neuroimage analyses from regional morphometry to networks connectivity” “networks” is modifying “connectivity” and as such should be written “network”
In line 393, “Although large-scale, multi-site studies have characterized age-related variations in brain morphology, microstructure, and networks connectivity, much of the findings stemmed from cross-sectional data; hence, limiting insights into true longitudinal changes.” This should be “Although large-scale, multi-site studies have characterized age-related variations in brain morphology, microstructure, and network connectivity, many of the findings stemmed from cross-sectional data, hence, limiting insights into true longitudinal changes.” [A semi-colon is not appropriate, as the phrase is not independent or complete.

In the final part of the section on Critical Gaps, the authors urge “longitudinal studies spanning from birth through late adulthood.” They are correct, but such studies can only be performed using staggered designs, as it is impractical and excessively slow to perform truly longitudinal studies given the urgency and the rate of change of imaging methodologies. I don’t think the authors have cited pediatric examples such as the longitudinal NKI Rockland Sample [https://pubmed.ncbi.nlm.nih.gov/35701428/] or the Chinese Color Nest Project [https://pubmed.ncbi.nlm.nih.gov/34653938/] which it inspired. A preprint describes the the 'Mapping Interindividual Variation in the Aging Connectome' (MIVAC) study which is an openly available resource relevant to aging https://pubmed.ncbi.nlm.nih.gov/41282656/.

Version 1:

Reviewer comments:

Reviewer #1

(Remarks to the Author)
The authors have satisfactorily addressed all my questions and concerns. I have no further comments and recommend the manuscript for publication.

Open Access This Peer Review File is licensed under a Creative Commons Attribution 4.0 International License, which permits use, sharing, adaptation, distribution and reproduction in any medium or format, as long as you give appropriate credit to the original author(s) and the source, provide a link to the Creative Commons license, and indicate if changes were made.
In cases where reviewers are anonymous, credit should be given to 'Anonymous Referee' and the source.
The images or other third party material in this Peer Review File are included in the article's Creative Commons license, unless indicated otherwise in a credit line to the material. If material is not included in the article's Creative Commons license and your intended use is not permitted by statutory regulation or exceeds the permitted use, you will need to obtain permission directly from the copyright holder.
To view a copy of this license, visit https://creativecommons.org/licenses/by/4.0/

Multiscale Brain Development Across the Human Lifespan: An MRI Perspective on Progress, Challenges, and Future Directions

Sahar Ahmad[1,2*] and Pew-Thian Yap[1,2*]

[1]Department of Radiology, The University of North Carolina at Chapel Hill, NC, USA.
[2]Biomedical Research Imaging Center (BRIC), The University of North Carolina at Chapel Hill, NC, USA.

*Corresponding author(s). E-mail(s): sahar967@email.unc.edu ptyap@med.unc.edu

Notes on revision

We would like to thank the editor and reviewers for their positive feedback and constructive comments. We have revised the review accordingly and highlighted major changes in *red*. Below, we provide detailed responses to the comments.

Response to the Reviewers

Reviewer 1 . . . 2
C1.1: Scope . . . 2
C1.2: Future Directions . . . 2
Reviewer 2 . . . 4
C2.1: Minor Points . . . 4

Reviewer 1

Summary: This is a timely and comprehensive review of lifespan brain development. The authors have done a commendable job synthesizing a vast literature across macrostructural, microstructural, and network scales, with particularly strong sections on morphometric similarity networks (MSNs) and structure-function coupling. The manuscript is logically structured, and the figures provide excellent orientation to the primary literature. However, before it is fully suitable for publication, there are a few conceptual and scoping issues that need to be addressed to enhance the manuscript's precision and theoretical depth.

C1.1: The title implies a modality-agnostic review of brain development, yet the content is strictly focused on magnetic resonance imaging (sMRI, dMRI, fMRI), which the authors appropriately note in their Keywords. However, the broader developmental connectomics field relies heavily on electrophysiological and optical imaging (MEG, EEG, fNIRS). This is especially critical at the early temporal extremes of the lifespan (neonates and infants), where MRI faces significant logistical and ethical constraints. The authors should either explicitly narrow the review's scope in the title (e.g., adding a subtitle like "An MRI Perspective") and introduction, or add a brief discussion acknowledging the complementary roles of these other modalities in filling the gaps left by MRI.

Thank you for the suggestion. We have changed the title and refined the language in the Abstract and Introduction to narrow the scope as follows:

Abstract: In this review, we synthesize current evidence to provide a multiscale *MRI-derived* account of structural and functional brain development across the human lifespan, highlight key conceptual and methodological gaps, and outline priorities for future research.

Introduction: In this review, we highlight current research on brain development across the lifespan, *emphasizing how different MRI modalities* capture neurodevelopmental processes at multiple spatial and temporal scales. We also discuss recent advances in computational methods that facilitate the processing and analysis of *brain MRIs*, thereby deepening our understanding of human brain development across the lifespan.

C1.2: While the perspectives section correctly identifies key future directions, it currently reads somewhat like a high-level checklist. Weaving in the following cutting-edge areas would significantly strengthen its theoretical and clinical impact:

Microstructure-to-Function Maturation: The manuscript thoroughly reviews microstructural changes (e.g., DTI/NODDI) and macro-level structure-function coupling, but the transition between these scales feels slightly disjointed. In the perspectives, it would be highly beneficial to discuss how specific micro-level alterations (e.g., neurite density, myelination trajectories) directly constrain or support the maturation of functional networks (e.g., DMN). This would better unify the multi-scale integration theme.

We have expanded the "Forward-Looking Perspectives" section as follows:

Lines 433–440: *Building upon previous works demonstrating that white matter microstructure provides a structural scaffold for the functional organization of large-scale brain networks [1–4], future research should expand these findings across the lifespan to establish mechanistic links between tissue microstructure and the emergence and development of functional networks. Integrating diffusion MRI or quantitative MRI measures to functional connectomics may elucidate how micro-level tissue properties, such as neurite density, myelin, tissue integrity, conduction velocity, etc., underlie network segregation and integration, and may also provide a basis to understand functional dysconnectivity in neurological conditions.*

Mechanisms of Individual Differences & Plasticity: Figure 4 excellently highlights "Genetics," "Environment," and "Social behavior." However, the accompanying text primarily frames these within the

context of “global sampling” and “demographic inclusivity.” The authors should expand on this to explicitly discuss how we can use multimodal lifespan data to understand the sources of individual variance and brain plasticity, moving beyond just having diverse samples to actually modeling the mechanistic impacts of genetics and early experiences.

Lines 425–430: *Future research in understanding brain plasticity will require large-scale epidemiologically informed studies that integrate MRI data with genetic, environmental, lifestyle, behavioral, and social measures across the lifespan [5, 6]. Multivariate analyses of these combined data will clarify how biological and social determinants interact to shape brain development and long-term health outcomes [7–9]. Such integrative approaches will help identify risk factors for cognitive decline and neurological diseases, and delineate age-specific windows of heightened vulnerability to these conditions over the lifetime.*

Bridging Methodological Advances (AI) with Clinical Translation: The authors highlight deep learning in the “Methodological Advances” section for image processing (e.g., skull-stripping, segmentation). They also end the review discussing the prediction of late-life neurodegenerative risk from early developmental deviations. These two themes should be connected. It would be worth noting how AI/deep learning could leverage these multi-scale normative atlases (like MSNs or SFC) to establish precise, personalized biological markers for the early identification of neurodevelopmental and neurodegenerative risks.

Lines 452–457: *In this context, deep learning models trained to capture lifelong SFC or dynamics of MSNs offer a predictive framework for the identification of brain disorders [10–13]. To this end, graph convolutional networks can integrate structural and functional connectivity, network topology, and nodal features to learn normative patterns of brain organization and the trained models can be used for disease classification [14, 15].*

Reviewer 2

Summary: This is an ambitious narrative review of brain development across the lifespan. The manuscript is well written and remarkably comprehensive. The encyclopedic review of the literature will serve as a resource for the developmental imaging community as it extends from the first decade of this century to recent milestones and fundamental methodological papers. The synthesis is brief but authoritative.

C2.1: A few minor points:

- In line 31, review does not need to be capitalized.
- In line 172, "In nutshell" should be "In a nutshell..."
- In line 278, "map" should be "maps"
- In line 349, "Latest work has introduced ..." is incomplete. It could be "The latest work..."
- In 359, the authors refer to gender. Gender, the psychological construct, is rarely the focus of neuroimaging studies, which instead dichotomize on biological sex.
- In 371, in "extending neuroimage analyses from regional morphometry to networks connectivity" "networks" is modifying "connectivity" and as such should be written "network"
- In line 393, "Although large-scale, multi-site studies have characterized age-related variations in brain morphology, microstructure, and networks connectivity, much of the findings stemmed from cross-sectional data; hence, limiting insights into true longitudinal changes." This should be "Although large-scale, multi-site studies have characterized age-related variations in brain morphology, microstructure, and network connectivity, many of the findings stemmed from cross-sectional data, hence, limiting insights into true longitudinal changes." [A semi-colon is not appropriate, as the phrase is not independent or complete.

We appreciate the reviewer's careful assessment and have made the suggested textual revisions accordingly.

- In the final part of the section on Critical Gaps, the authors urge "longitudinal studies spanning from birth through late adulthood." They are correct, but such studies can only be performed using staggered designs, as it is impractical and excessively slow to perform truly longitudinal studies given the urgency and the rate of change of imaging methodologies. I don't think the authors have cited pediatric examples such as the longitudinal NKI Rockland Sample [https://pubmed.ncbi.nlm.nih.gov/35701428/] or the Chinese Color Nest Project [https://pubmed.ncbi.nlm.nih.gov/34653938/] which it inspired. A preprint describes the 'Mapping Interindividual Variation in the Aging Connectome' (MIVAC) study which is an openly available resource relevant to aging https://pubmed.ncbi.nlm.nih.gov/41282656/.

These studies have now been cited in the "Forward-Looking Perspectives" section as follows:

Lines 410–415: *Recent large-scale initiatives, such as the HEALthy Brain and Child Development (HBCD) study [16], the Adolescent Brain Cognitive Development (ABCD) study [17], the NKI Rockland Sample [18], the Mapping Interindividual Variation in the Aging Connectome (MIVAC) study [19], and the Chinese Color Nest Project [20], provide unprecedented opportunities for characterizing within-individual changes in brain structure and function, extending the knowledge gained from cross-sectional data.*

References

[1] Gordon, E.M., Lee, P.S., Maisog, J.M., Foss-Feig, J., Billington, M.E., VanMeter, J., Vaidya, C.J.: Strength of default mode resting-state connectivity relates to white matter integrity in children. Developmental Science **14**(4), 738–751 (2010) https://doi.org/10.1111/j.1467-7687.2010.01020.x

[2] Teipel, S.J., Bokde, A.L.W., Meindl, T., Amaro, E., Soldner, J., Reiser, M.F., Herpertz, S.C., Möller, H.-J., Hampel, H.: White matter microstructure underlying default mode network connectivity in the human brain. NeuroImage **49**(3), 2021–2032 (2010) https://doi.org/10.1016/j.neuroimage.2009.10.067

[3] Nelson, M.C., Da Lu, W., Leppert, I.R., Hansen, H.A., Rowley, C.D., Misic, B., Tardif, C.L.: The role of white matter myelin in structural-functional network coupling. Communications Biology **9**(1) (2026) https://doi.org/10.1038/s42003-026-09813-6

[4] Park, B.-y., Paquola, C., Bethlehem, R.A.I., Benkarim, O., Mišić, B., Smallwood, J., Bullmore, E.T., Bernhardt, B.C., Bullmore, E., Dolan, R., Goodyer, I., Fonagy, P., Jones, P., Moutoussis, M., Hauser, T., Neufeld, S., Romero-Garcia, R., St Clair, M., Vértes, P., Whitaker, K., Inkster, B., Prabhu, G., Ooi, C., Toseeb, U., Widmer, B., Bhatti, J., Villis, L., Alrumaithi, A., Birt, S., Bowler, A., Cleridou, K., Dadabhoy, H., Davies, E., Firkins, A., Granville, S., Harding, E., Hopkins, A., Isaacs, D., King, J., Kokorikou, D., Maurice, C., McIntosh, C., Memarzia, J., Mills, H., O'Donnell, C., Pantaleone, S., Scott, J., Kiddle, B., Polek, E., Fearon, P., Suckling, J., Harmelen, A.-L., Kievit, R., Chamberlain, S.: Adolescent development of multiscale structural wiring and functional interactions in the human connectome. Proceedings of the National Academy of Sciences **119**(27) (2022) https://doi.org/10.1073/pnas.2116673119

[5] Walhovd, K.B., Fjell, A.M., Westerhausen, R., Nyberg, L., Ebmeier, K.P., Lindenberger, U., Bartres-Faz, D., Baare, W.F.C., Siebner, H.R., Henson, R., Drevon, C.A., Knudsen, G.P., Budin-Ljøsne, I., Penninx, B.W.J.H., Ghisletta, P., Rogeberg, O., Tyler, L., Bertram, L.a.: Healthy minds 0–100 years: Optimising the use of European brain imaging cohorts ("Lifebrain"). European Psychiatry **47**, 76–77 (2018) https://doi.org/10.1016/j.eurpsy.2017.10.005

[6] Dick, A.S., Lopez, D.A., Watts, A.L., Heeringa, S., Reuter, C., Bartsch, H., Fan, C.C., Kennedy, D.N., Palmer, C., Marshall, A., Haist, F., Hawes, S., Nichols, T.E., Barch, D.M., Jernigan, T.L., Garavan, H., Grant, S., Pariyadath, V., Hoffman, E., Neale, M., Stuart, E.A., Paulus, M.P., Sher, K.J., Thompson, W.K.: Meaningful associations in the adolescent brain cognitive development study. NeuroImage **239**, 118262 (2021) https://doi.org/10.1016/j.neuroimage.2021.118262

[7] Modabbernia, A., Janiri, D., Doucet, G.E., Reichenberg, A., Frangou, S.: Multivariate patterns of brain-behavior-environment associations in the adolescent brain and cognitive development study. Biological Psychiatry **89**(5), 510–520 (2021) https://doi.org/10.1016/j.biopsych.2020.08.014

[8] Brouwer, R.M., Klein, M., Grasby, K.L., Schnack, H.G., Jahanshad, N., Teeuw, J., Thomopoulos, S.I., Sprooten, E., Franz, C.E., Gogtay, N., Kremen, W.S., Panizzon, M.S., Olde Loohuis, L.M., Whelan, C.D., Aghajani, M., Alloza, C., Alnæs, D., Artiges, E., Ayesa-Arriola, R., Barker, G.J., Bastin, M.E., Blok, E., Bøen, E., Breukelaar, I.A., Bright, J.K., Buimer, E.E.L., Bülow, R., Cannon, D.M., Ciufolini, S., Crossley, N.A., Damatac, C.G., Dazzan, P., Mol, C.L., Zwarte, S.M.C., Desrivières, S., Díaz-Caneja, C.M., Doan, N.T., Dohm, K., Fröhner, J.H., Goltermann, J., Grigis, A., Grotegerd, D., Han, L.K.M., Harris, M.A., Hartman, C.A., Heany, S.J., Heindel, W., Heslenfeld, D.J., Hohmann, S., Ittermann, B., Jansen, P.R., Janssen, J., Jia, T., Jiang, J., Jockwitz, C., Karali, T., Keeser, D., Koevoets, M.G.J.C., Lenroot, R.K., Malchow, B., Mandl, R.C.W., Medel, V., Meinert, S., Morgan, C.A., Mühleisen, T.W., Nabulsi, L., Opel, N., Foz, V.O.-G., Overs, B.J., Paillère Martinot, M.-L., Redlich, R., Marques, T.R., Repple, J., Roberts, G., Roshchupkin, G.V., Setiaman, N., Shumskaya, E., Stein, F., Sudre, G., Takahashi, S., Thalamuthu, A., Tordesillas-Gutiérrez, D., Lugt, A., Haren, N.E.M., Wardlaw, J.M., Wen, W., Westeneng, H.-J., Wittfeld, K., Zhu, A.H., Zugman, A., Armstrong, N.J., Bonfiglio, G., Bralten, J., Dalvie, S., Davies, G., Di Forti, M., Ding, L., Donohoe, G., Forstner, A.J., Gonzalez-Peñas, J., Guimaraes, J.P.O.F.T., Homuth, G., Hottenga, J.-J., Knol, M.J., Kwok, J.B.J., Le Hellard, S., Mather, K.A.,

Milaneschi, Y., Morris, D.W., Nöthen, M.M., Papiol, S., Rietschel, M., Santoro, M.L., Steen, V.M., Stein, J.L., Streit, F., Tankard, R.M., Teumer, A., Ent, D., Meer, D., Eijk, K.R., Vassos, E., Vázquez-Bourgon, J., Witt, S.H., Brühl, R., Papadopoulos Orfanos, D., Paus, T., Millenet, S., Adams, H.H.H., Agartz, I., Ames, D., Amunts, K., Andreassen, O.A., Arango, C., Banaschewski, T., Baune, B.T., Belangero, S.I., Bokde, A.L.W., Boomsma, D.I., Bressan, R.A., Brodaty, H., Buitelaar, J.K., Cahn, W., Caspers, S., Cichon, S., Crespo-Facorro, B., Cox, S.R., Dannlowski, U., Elvsåshagen, T., Espeseth, T., Falkai, P.G., Fisher, S.E., Flor, H., Fullerton, J.M., Garavan, H., Gowland, P.A., Grabe, H.J., Hahn, T., Heinz, A., Hillegers, M., Hoare, J., Hoekstra, P.J., Ikram, M.A., Jackowski, A.P., Jansen, A., Jönsson, E.G., Kahn, R.S., Kircher, T., Korgaonkar, M.S., Krug, A., Lemaitre, H., Malt, U.F., Martinot, J.-L., McDonald, C., Mitchell, P.B., Muetzel, R.L., Murray, R.M., Nees, F., Nenadić, I., Oosterlaan, J., Ophoff, R.A., Pan, P.M., Penninx, B.W.J.H., Poustka, L., Sachdev, P.S., Salum, G.A., Schofield, P.R., Schumann, G., Shaw, P., Sim, K., Smolka, M.N., Stein, D.J., Trollor, J.N., Berg, L.H., Veldink, J.H., Walter, H., Westlye, L.T., Whelan, R., White, T., Wright, M.J., Medland, S.E., Franke, B., Thompson, P.M., Hulshoff Pol, H.E.: Genetic variants associated with longitudinal changes in brain structure across the lifespan. Nature Neuroscience **25**(4), 421–432 (2022) https://doi.org/10.1038/s41593-022-01042-4

[9] Modabbernia, A., Reichenberg, A., Ing, A., Moser, D.A., Doucet, G.E., Artiges, E., Banaschewski, T., Barker, G.J., Becker, A., Bokde, A.L.W., Quinlan, E.B., Desrivières, S., Flor, H., Fröhner, J.H., Garavan, H., Gowland, P., Grigis, A., Grimmer, Y., Heinz, A., Insensee, C., Ittermann, B., Martinot, J.-L., Martinot, M.-L.P., Millenet, S., Nees, F., Orfanos, D.P., Paus, T., Penttilä, J., Poustka, L., Smolka, M.N., Stringaris, A., Noort, B.M., Walter, H., Whelan, R., Schumann, G., Frangou, S.a.: Linked patterns of biological and environmental covariation with brain structure in adolescence: a population-based longitudinal study. Molecular Psychiatry **26**(9), 4905–4918 (2020) https://doi.org/10.1038/s41380-020-0757-x

[10] Zhang, L., Wang, L., Gao, J., Risacher, S.L., Yan, J., Li, G., Liu, T., Zhu, D.: Deep fusion of brain structure-function in mild cognitive impairment. Medical Image Analysis **72**, 102082 (2021) https://doi.org/10.1016/j.media.2021.102082

[11] Xia, J., Chan, Y.H., Girish, D., Rajapakse, J.C.: Interpretable modality-specific and interactive graph convolutional network on brain functional and structural connectomes. Medical Image Analysis **102**, 103509 (2025) https://doi.org/10.1016/j.media.2025.103509

[12] Park, H.W., Lee, W.H.: Morphometric similarity network-based graph convolutional networks for schizophrenia classification. Scientific Reports **15**(1) (2025) https://doi.org/10.1038/s41598-025-19894-8

[13] Yang, Y., Ye, C., Guo, X., Wu, T., Xiang, Y., Ma, T.: Mapping multi-modal brain connectome for brain disorder diagnosis via cross-modal mutual learning. IEEE Transactions on Medical Imaging **43**(1), 108–121 (2024) https://doi.org/10.1109/tmi.2023.3294967

[14] Park, H.W., Kim, S.Y., Lee, W.H.: Graph Convolutional Network with Morphometric Similarity Networks for Schizophrenia Classification, pp. 626–636. Springer, Switzerland (2023). https://doi.org/10.1007/978-3-031-43907-0_60

[15] Sunil, G., Gowtham, S., Bose, A., Harish, S., Srinivasa, G.: Graph neural network and machine learning analysis of functional neuroimaging for understanding schizophrenia. BMC Neuroscience **25**(1) (2024) https://doi.org/10.1186/s12868-023-00841-0

[16] Volkow, N.D., Gordon, J.A., Bianchi, D.W., Chiang, M.F., Clayton, J.A., Klein, W.M., Koob, G.F., Koroshetz, W.J., Pérez-Stable, E.J., Simoni, J.M., Tromberg, B.J., Woychik, R.P., Hommer, R., Spotts, E.L., Xu, B., Zehr, J.L., Cole, K.M., Dowling, G.J., Freund, M.P., Howlett, K.D., Jordan, C.J., Murray, T.M., Pariyadath, V., Prabhakar, J., Rankin, M.L., Sarampote, C.S., Weiss, S.R.B.: The HEALthy Brain and Child Development Study (HBCD): NIH collaboration to understand the impacts of prenatal and early life experiences on brain development. Developmental Cognitive Neuroscience **69**, 101423 (2024) https://doi.org/10.1016/j.dcn.2024.101423

[17] Volkow, N.D., Koob, G.F., Croyle, R.T., Bianchi, D.W., Gordon, J.A., Koroshetz, W.J., Pérez-Stable, E.J., Riley, W.T., Bloch, M.H., Conway, K., Deeds, B.G., Dowling, G.J., Grant, S., Howlett, K.D., Matochik, J.A., Morgan, G.D., Murray, M.M., Noronha, A., Spong, C.Y., Wargo, E.M., Warren, K.R., Weiss, S.R.B.: The conception of the ABCD study: From substance use to a broad NIH collaboration. Developmental Cognitive Neuroscience **32**, 4–7 (2018) https://doi.org/10.1016/j.dcn.2017.10.002

[18] Tobe, R.H., MacKay-Brandt, A., Lim, R., Kramer, M., Breland, M.M., Tu, L., Tian, Y., Trautman, K.D., Hu, C., Sangoi, R., Alexander, L., Gabbay, V., Castellanos, F.X., Leventhal, B.L., Craddock, R.C., Colcombe, S.J., Franco, A.R., Milham, M.P.: A longitudinal resource for studying connectome development and its psychiatric associations during childhood. Scientific Data **9**(1) (2022) https://doi.org/10.1038/s41597-022-01329-y

[19] MacKay-Brandt, A., Gazes, Y., Garcia-Barnett, D., Grebe, L., Ripley, O., Gan, K.X., Trautman, K.T., Kramer, M., Breland, M., Tobe, R., Franco, A., Gabbay, V., Milham, M., Colcombe, S.: An open, longitudinal resource for mapping interindividual variation in the aging connectome (2025) https://doi.org/10.1101/2025.10.10.25337774

[20] Liu, S., Wang, Y.-S., Zhang, Q., Zhou, Q., Cao, L.-Z., Jiang, C., Zhang, Z., Yang, N., Dong, Q., Zuo, X.-N.: Chinese Color Nest Project: An accelerated longitudinal brain-mind cohort. Developmental Cognitive Neuroscience **52**, 101020 (2021) https://doi.org/10.1016/j.dcn.2021.101020
